# Analysis of the Maturation of the Median Nerve in Preterm‐Born Children During the First 3 Years of Life Using High‐Resolution Nerve Ultrasound Imaging

**DOI:** 10.1002/brb3.70954

**Published:** 2025-10-01

**Authors:** Lynn Jansen, Noé Bürke, Erin West, Janina Wurster, Philip J. Broser

**Affiliations:** ^1^ Medical Faculty University of St. Gallen and Zurich St. Gallen Switzerland; ^2^ Clinical Trials Unit Health Ostschweiz (Hoch) St. Gallen Switzerland; ^3^ Neuropediatrics Children's Hospital of Eastern Switzerland St. Gallen Switzerland

**Keywords:** high‐resolution ultrasound imaging, maturation of the peripheral nervous system, myelination, prematurity

## Abstract

**Aim:**

To compare the development of the peripheral nervous systems of preterm‐ and term‐born children from birth to 3 years of age by imaging the median nerve.

**Methods:**

Measuring the cross‐sectional area (CSA) of the median nerve at three locations along the arm in term‐born (control group) and preterm‐born children (study group) using high‐resolution ultrasound imaging.

**Results:**

The data revealed a steady myelination that follows a logarithmic curve when considering the increase of the CSA of the median nerve relative to body surface area (BSA) in preterm‐born children. Therefore, while the CSA of the median nerve was smaller in premature babies at the time of birth, the maturation of the nerve is comparable for preterm‐ and term‐born children.

**Interpretation:**

This study analyzed how the peripheral nervous system of preterm children develops compared to full‐term children and found no differences in terms of median nerve development. Further, these findings align with previous research documenting a logarithmic increase in the CSA of the median nerve with age in full‐term infants. This research could be helpful for enabling the use of high‐resolution ultrasound imaging as a diagnostic tool for the peripheral nervous system of premature infants.

## Graphical abstract

High‐resolution ultrasound imaging was used to examine the median nerve of preterm‐ and term‐born children. In both groups, the cross‐sectional area of the median nerve increases logarithmically with body‐surface area, and statistical tests show no difference, suggesting similar development of the peripheral nervous system.

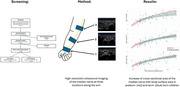

**Abbreviations**
APGAR: appearance, pulse, grimace, activity, and respiration
BSA: body surface area
CSA: cross‐sectional area
NCV: nerve conduction velocity

## 1 Introduction

The chances of survival for premature infants, especially extremely premature ones, have significantly improved in recent years (Bell et al. 2022; Cao et al. 2022; Stoll et al. 2015), which can be partly attributed to better medical care (Zeitlin et al. 2016). However, it is widely known that preterm‐born children are at much higher risk for neurodevelopmental and neurobehavioral problems. For instance, they exhibit impaired motor function, including issues with balance as well as fine and gross motor skills (De Kieviet et al. 2009; Luu et al. 2017; R. Pineda et al. 2022; R. G. Pineda et al. 2013). While the development of the central nervous system in preterm‐born children has been thoroughly studied (Doria et al. 2014; Sie et al. 1997; Van de Bor et al. 1990), there is limited research on the peripheral nervous system for this group.

Myelination of the peripheral nervous system is one of the most crucial processes during postnatal maturation. The first 2 years of life are particularly critical for the development of the myelin sheath of the peripheral nerve. During this time, nerve conduction velocity (NCV) roughly doubles (Ryan et al. 2019). This increase in NCV goes hand in hand with the increase in the cross‐sectional area (CSA) of the nerve, and both these processes follow a logarithmic curve (Jenny et al. 2020). It is not known whether this increase in the CSA also appears in children who are born prematurely. However, some of the motor problems seen in premature infants may be caused by delayed myelination.

Considerable research has been conducted on the peripheral nerves in older children (Cartwright et al. 2013, 2008; Druzhinin et al. 2019; Schubert et al. 2020). The NCV of peripheral nerves in premature infants has been measured in some studies, but their results are inconsistent (Lori et al. 2018; Smit et al. 1999; Tombini et al. 2009; Tranier et al. 1989).

Although previous works have focused on examining the development of the peripheral nervous system in preterm‐born children via NCV, ultrasound imaging could provide additional insights while greatly reducing the burden for the children. One of the challenges due to which this examination method has not yet been used for premature babies is that the main peripheral nerves of neonates are extremely small (i.e., the median nerve has a CSA of hardly 1.5 mm^2^; Jenny et al. 2020), making them difficult to measure with standard ultrasound methods. Modern high‐frequency ultrasound probes (20 MHz) have made it possible to image small structures and measure the CSA of the median nerve in such children.

This method has recently been used to study the development of the peripheral nervous system in term‐born children who were younger than 1 year. In these studies, similar results of a logarithmic increase in the CSA of the nerves with age were found for the median nerve, the nervus ischiadicus, and the nerve roots (Jenny et al. 2020, 2023; Van der Linde et al. 2022).

The current study aims to analyze the maturation of the peripheral nervous system in premature infants using high‐resolution ultrasound imaging. Given that two‐thirds of the myelination occurs during the first 2 years of life (Jenny et al. 2020), the age range considered in this study was from birth to 3 years of age. The median nerve has been used for measurements in previous studies because it is easily accessible for ultrasound imaging and can be measured with high reproducibility, which makes it well suited for monitoring the maturation of the peripheral nervous system. This nerve was systematically imaged in preterm‐ and term‐born children at three locations (wrist, forearm, and upper arm), as done in previous research (Jenny et al. 2020).

## 2 Methods

This cross‐sectional study was conducted at the Eastern Children's Hospital in St. Gallen, Switzerland, from March 2023 to May 2025. Ethical approval was granted, and the project was registered in the Swiss project database (Basec 2019‐02200, EKOS 19/166). All guardians consented to have their child included in this study.

Children who were hospitalized due to premature birth or who were undergoing development assessments due to prematurity were screened for the study group. Those hospitalized due to infections, orthopedic problems, or other medical conditions were evaluated for both the study and the control group.

The inclusion criterion for both groups was an age range of birth to 3 years. Patients with a family history of inherited diseases, genetic or developmental abnormalities, syndromes, and neuropathy were excluded from the study.

The study group included only preterm‐born children (World Health Organization 1975). The control group included children from Gestational Weeks 36 to 42.

**FIGURE 1:**
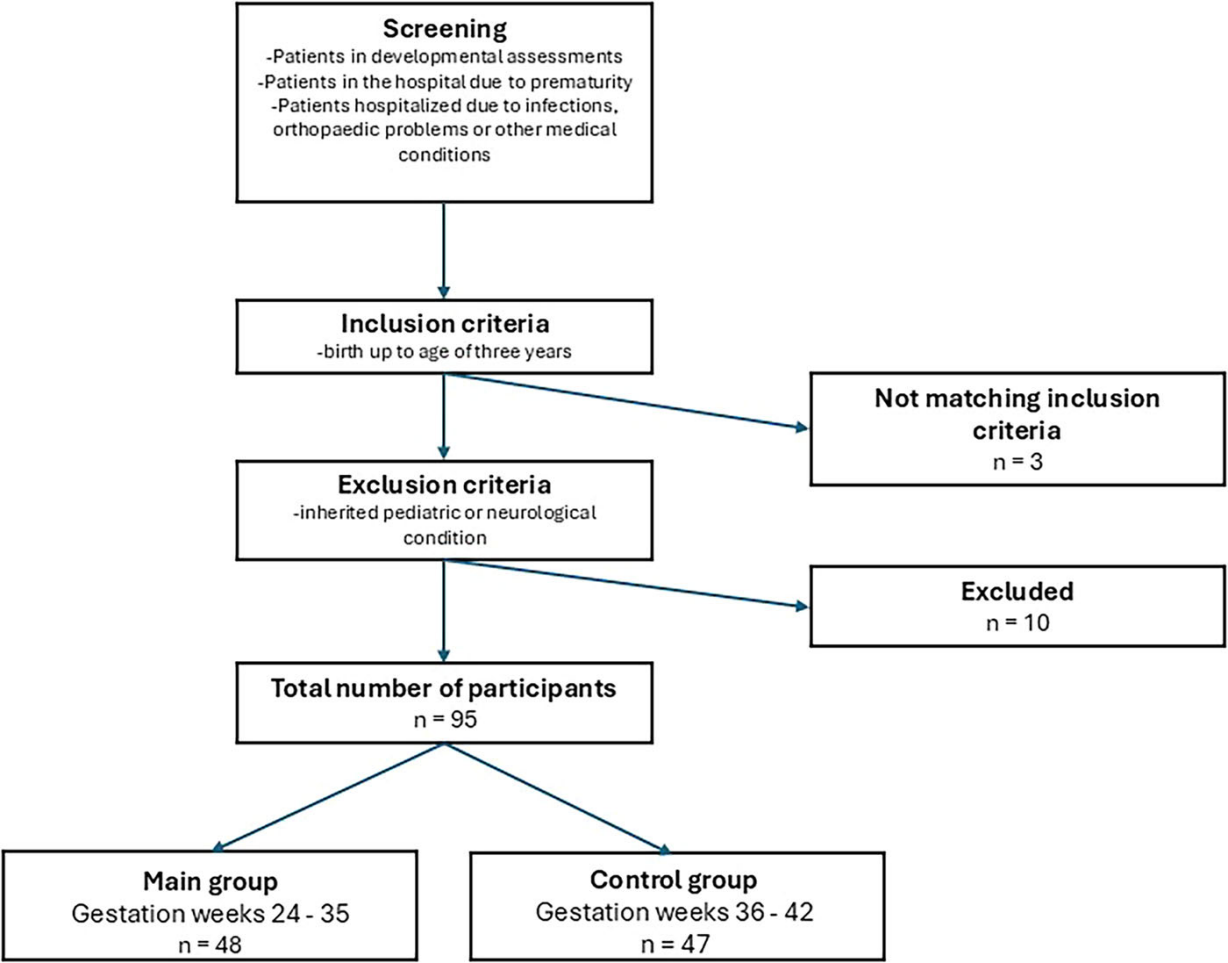
Flowchart of the inclusion and exclusion criteria with the final study and control group.

A total of 108 patients were screened for inclusion in this study. Three did not meet the inclusion criteria because they were over 3 years old, and ten were excluded for medical reasons. Ultimately, a total of 95 patients were included in the analysis: 48 in the study group and 47 in the control group (Figure 1). The age range of the study group participants was from 6 days to 973 days, with corrected ages ranging from ‐73 to 910 days. The baby born at the earliest gestational age was born at 25 weeks. The age range of the control group participants was from 9 days to 1080 days.Supporting Information

The ultrasound examinations were performed at each child's bedside or, for older patients, in the examination room. In all cases, a child‐friendly atmosphere was established prior to conducting the examination.

All examinations were performed using a Canon Aplio i800 ultrasound imaging system (Canon Aplio I‐Series). The main probe used was the i22LH8 22 MHz ultra‐high‐frequency linear transducer, which has been previously tested and shown to reliably measure nerves in newborns (Jenny et al. 2020).

**FIGURE 2:**
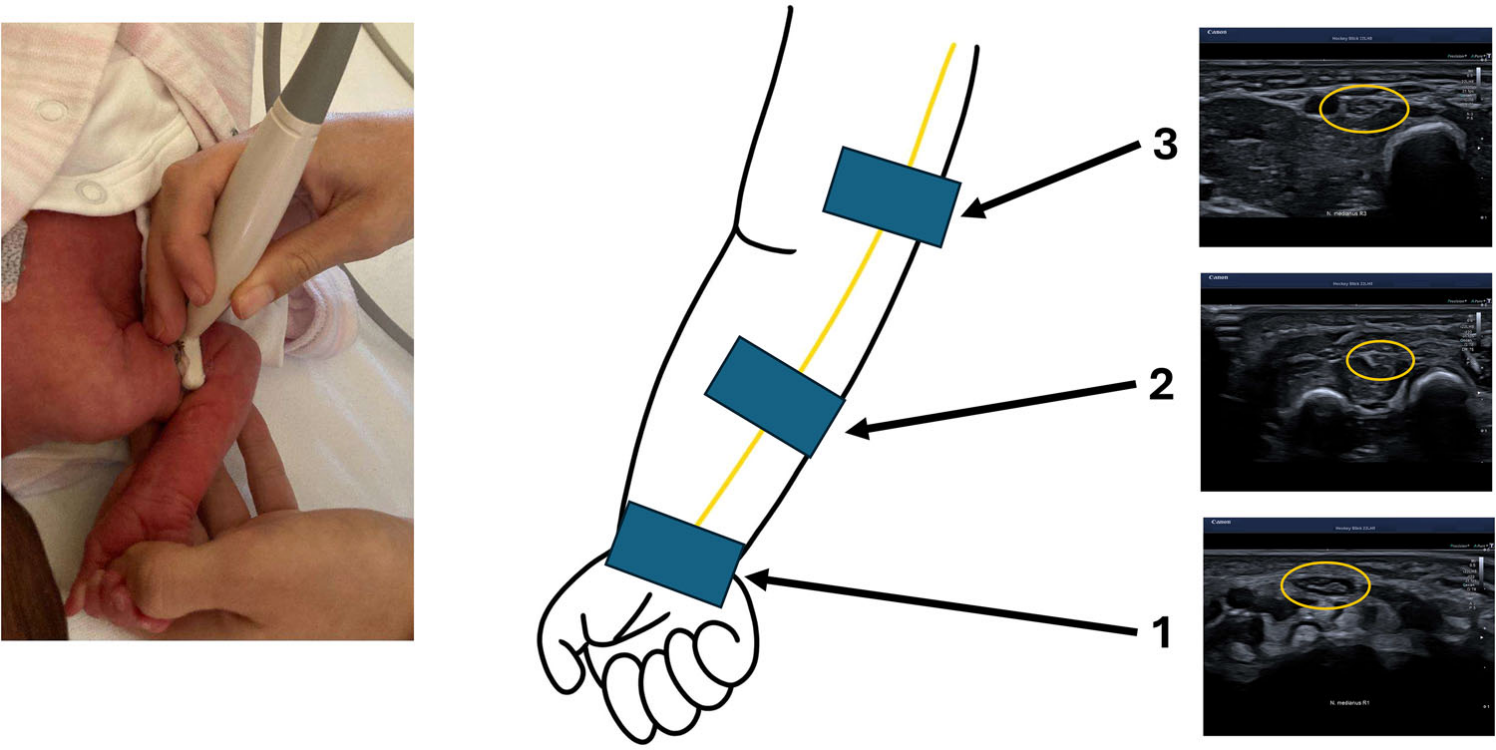
Demonstration of the high‐resolution ultrasound imaging of the median nerve at the three locations and ultrasound images of the median nerve in cross‐section.

Ultrasound images of the median nerve were taken at three locations on both arms. At each location, the nerve was displayed with a transverse orthogonal view, as described by Gruber and Peer (2013); also see Gruber et al. (2018). Figure 2 illustrates the locations at which the nerve was measured.

Location 1 refers to the spot just before the nerve enters the carpal tunnel at the wrist level. Location 2 is in the middle of the forearm, where the median nerve is embedded between the superficial and deep hand flexor muscles. Finally, Location 3 is situated at the level of the lower upper arm. Here, the nerve was imaged next to the brachial artery at the medial aspect of the arm, and a Doppler ultrasound was used to better differentiate between the nerve and brachial artery.

The procedure followed here is described in more detail by Jenny et al. (2020). When possible, the examination was repeated on the other arm. If medical devices, such as an intravenous catheter or cast, obstructed the view, or if the child was no longer compliant, not all locations were imaged. If a nerve location was not easily visible, a short video was recorded for subsequent processing.

To reduce the examination time, all nerve tracing and measurements were performed after the ultrasound imaging using either still images or video frames. For all three locations and both arms, the freehand tracing tool of the ultrasound imaging machine was used to trace the nerve. After tracing, the ultrasound machine calculated the CSA and circumference of the nerve in square millimeters and millimeters. These values were recorded along with the patients’ gestational weeks, birth dates, sexes, heights, weights, and clinical data in an Excel spreadsheet using the hospital computer. Anonymized data are available upon request.

Sonography was performed by N.B., J.W., and L.J. and supervised by P.B. N.B. and L.J. carried out the measurements strictly as outlined above. Intraclass correlation coefficients were calculated for reviewers N.B. and L.J. using images of Area 2 of the arm of eleven patients to test measurement reliability between raters.

All statistical calculations were performed using R version 4.3.2 (R Core Team n.d.). For the main analysis, the participants were divided into two groups: (1) children born prematurely in Weeks 24–35 of gestation and (2) children born at term in Weeks 36–42 of gestation. If no data on the gestational week at birth were available, it was assumed that the baby was born at 40 weeks. Since the nerve's CSA was measured on both the right and the left arm, the mean of the measurements was used when data for both sides were available. If data were available only for one arm, the CSA measurement for that side was used. Body surface area (BSA) was calculated using Meban's formula (Ahn 2010; Ahn and Garruto 2008). Further, the percentiles of length and weight were calculated using data from the Children's Hospital Zurich, as they had data that would allow for the calculation of the percentiles for premature infants (Braegger et al. 2011). To compare ages between the study and control groups, conceptual age was used.

Descriptive statistics for the participants are presented as means and standard deviations for continuous variables and as frequencies and percentages for categorical variables.

To determine the comparability of the two reviewers’ measurements, the intraclass correlation coefficient was calculated for the CSA and circumference of the median nerve. Accordingly, Location 2 (forearm) on the left side was measured by both reviewers for eleven patients.

Scatterplots of the CSA and BSA values were generated to illustrate the relationship between the two with an increase in BSA for both term‐ and preterm‐born infants.

Further, gestational age and BSA were tested for interaction terms, and no collinearity was found. We then tested whether continuous BSA orage from conception (Supplementary 1, Supplementary 2) better predicted CSA and found that BSA was the stronger predictor. Linear regression models of CSA were created for all three locations, and their relationship with log‐transformed BSA and gestational age group (preterm‐ and term‐born age groups) was assessed. Therefore, log‐transformed BSA was used, as previous research has shown that increases in CSA follow a logarithmic curve (Jenny et al. 2020). These models were used to understand how CSA growth is related to BSA and gestational age group.

To account for multiple comparisons, the Benjamini–Hochberg procedure was used (Benjamini and Hochberg 1995). A false discovery rate of 0.05 was assumed, and eight statistical tests were evaluated.

## 3 Results

The demographics of the participants are presented in Table 1. To account for multiple comparisons, the Benjamini–Hochberg procedure was implemented for the results of eight statistical tests to control the false discovery rate of 0.05. Five tests remained statistically significant after this correction, with only one *p* < 0.05 losing statistical significance.

**TABLE 1:** Demographics by group.

	**Preterm‐born**	**Term‐born**	**Overall**
	**(*N *= 48)**	**(*N *= 47)**	**(*N *= 95)**
Age from conception (in days)			
Mean (SD)	420 (272)	594 (325)	506 (311)
Median [min, max]	275 [207, 1190]	464 [289, 1360]	365 [207, 1360]
Sex			
Female	22 (45.8%)	21 (44.7%)	43 (45.3%)
Male	26 (54.2%)	26 (55.3%)	52 (54.7%)
Weight Percentile			
Mean (SD)	58.1 (38.2)	48.6 (34.6)	53.4 (36.6)
Median [min, max]	65.1 [1.00, 99.0]	47.8 [0, 99.2]	53.2 [0, 99.2]
Missing	1 (2.1%)	1 (2.1%)	2 (2.1%)
Length percentile			
Mean (SD)	52.5 (44.2)	50.1 (32.9)	51.4 (39.3)
Median [min, max]	50.0 [1.00, 99.0]	48.3 [0, 100]	49.2 [0, 100]
Meban BSA			
Mean (SD)	56.4 (27.5)	76.4 (25.1)	65.5 (28.1)
Median [min, max]	41.2 [22.3, 118]	67.3 [38.4, 122]	59.7 [22.3, 122]
Missing	1 (2.1%)	8 (17.0%)	9 (9.5%)
Area 1			
Mean (SD)	2.38 (0.968)	2.74 (0.682)	2.55 (0.863)
Median [min, max]	2.11 [0.870, 4.63]	2.88 [1.37, 3.77]	2.46 [0.870, 4.63]
Missing	1 (2.1%)	7 (14.9%)	8 (8.4%)
Area 2			
Mean (SD)	2.23 (0.852)	2.67 (0.767)	2.43 (0.838)
Median [min, max]	2.08 [0.790, 4.34]	2.60 [1.05, 4.84]	2.39 [0.790, 4.84]
Missing	0 (0%)	4 (8.5%)	4 (4.2%)
Area 3			
Mean (SD)	2.61 (0.937)	2.98 (0.744)	2.78 (0.868)
Median [min, max]	2.41 [0.940, 5.12]	2.89 [1.80, 4.90]	2.60 [0.940, 5.12]
Missing	1 (2.1%)	6 (12.8%)	7 (7.4%)

The intraclass correlation coefficients for the two reviewers were found to be 0.994 and 0.992 (both *p* < 0.001) for area and circumference, respectively (Table 2). This indicated that there was adequate comparability between the two reviewers’ measurements.

**TABLE 2:** Intraclass correlation analyses of the two reviewers for Location 2 on the left.

Variable	intraclass correlation coefficient	95% confidence interval	*p* value		Sample size
L2 Area	0.994	0.977–0.998	< 0.001		11
L2 Circ	0.992	0.971–0.998	< 0.001		11

**FIGURE 3:**
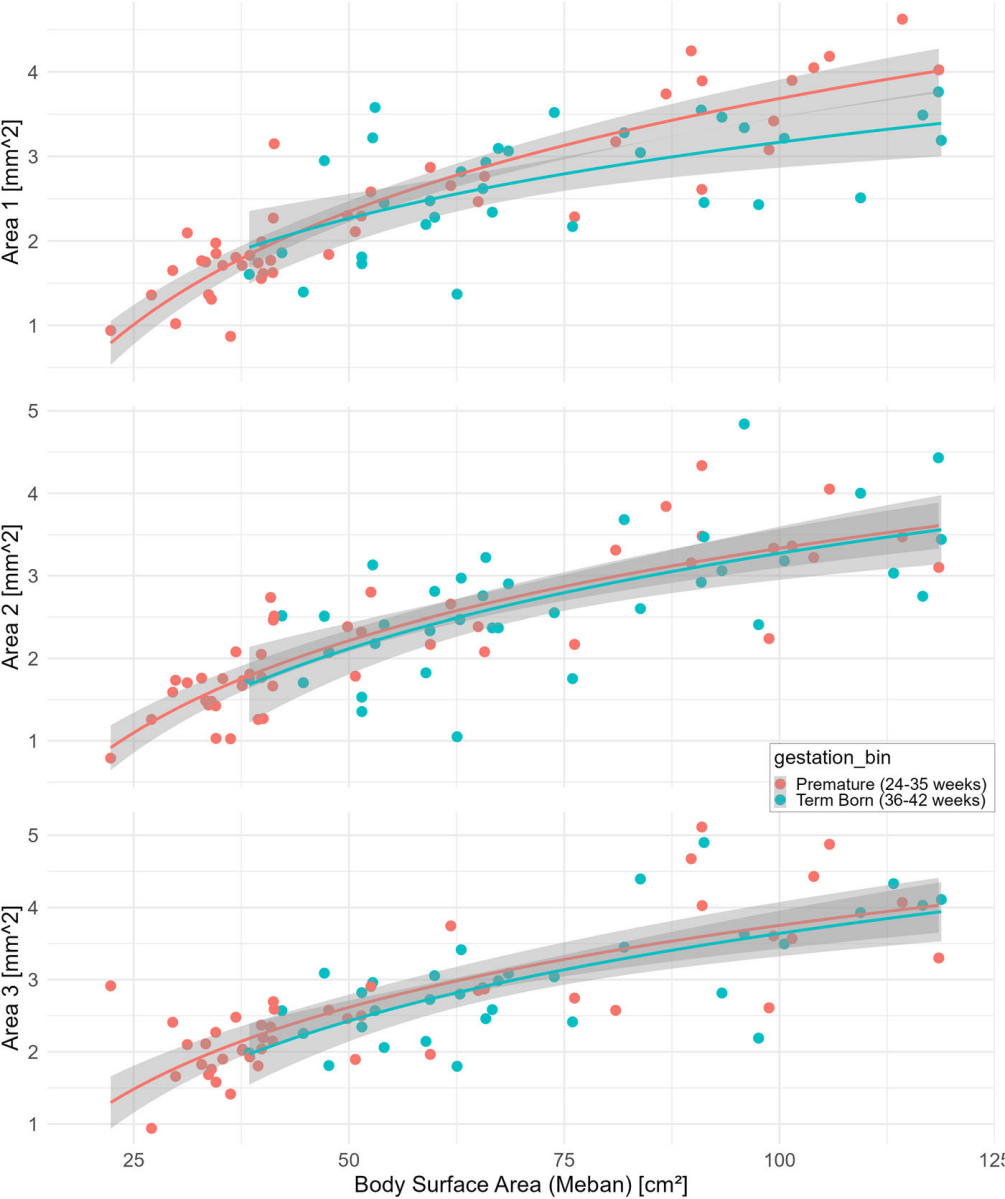
Increase of CSA with BSA in premature (red) and full‐term (blue) infants for all locations (wrist, forearm, and upper arm).

Figure 3 shows scatterplots of CSA in relation to BSA, demonstrating the nonlinear increase in CSA with BSA. In premature infants, the CSA is much smaller at the time of birth compared to full‐term infants. For both groups, steady myelination can be seen with an increase in BSA, as illustrated by the logarithmic regression line. The regressions of the CSA with the BSA of the preterm and full‐term children overlap. A slightly higher CSA with higher BSA was detected only at Location 1 (level of the wrist).

The linear models of CSA (Table 3) show statistically significant *p* values of less than 0.001 for log‐transformed BSA, suggesting a high influence of BSA on CSA (log‐transformed BSA: Area 1: *p* < 0.001, Estimate = 1.762; Area 2: *p* < 0.001, Estimate = 1.606; Area 3: *p* < 0.001, Estimate = 1.654). Gestational age group was found to be less influential on CSA (gestational age group: Area 1: *p* = 0.041, Estimate = 0.247; Area 2: *p* = 0.473, Estimate = 0.091; Area 3: *p* = 0.235, Estimate = 0.167), with only Area 1 having a *p* value of less than 0.05. However, this was not statistically significant due to the multiple comparison adjustment.

**TABLE 3:** Models of CSA against log‐transformed BSA and gestational age for all three locations.

**Outcome**	**Predictor**	**Estimate**	**95% CI**	***p* value**
				
CSA 1	Log BSA (Meban)	1.762	[1.493, 2.032]	< 0.001
CSA 1	Gestational age group (premature)	0.247	[0.011, 0.484]	0.041
CSA 2	Log BSA (Meban)	1.606	[1.319, 1.893]	< 0.001
CSA 2	Gestational age group (premature)	0.091	[−0.159, 0.341]	0.473
CSA 3	Log BSA (Meban)	1.654	[1.333, 1.974]	< 0.001
CSA 3	Gestational age group (premature)	0.167	[−0.111, 0.444]	0.235

## 4 Discussion

This study compared the development of the peripheral nervous system between premature and full‐term infants by measuring the CSA of the median nerve at three locations. A total of 48 preterm‐ and 47 term‐born children were analyzed.

In the linear models, we found log‐transformed BSA to be a statistically significant predictor of CSA (Table 2). Gestational age group was not a predictor of CSA, suggesting that the CSA of premature infants does not develop differently from their full‐term counterparts. The scatterplots visually confirm these findings. This suggests that the peripheral nervous system of preterm‐born children develops in the same manner as that of term‐born children.

Histopathological studies found similar results. Schröder et al. (1978) showed an increase in myelin thickness in the sural nerve, which is initially more rapid during the fetal period and slows with age, resembling a logarithmic regression line. Another study examined vagal nerve maturation in lambs during the fetal period, finding no myelination during the first weeks of gestation, followed by a rapid increase in myelin thickness (Hasan et al. 1993).

Further, our findings align with those of Jenny et al. (2020). Thus, these logarithmically transformed linear models for CSA may also be used for premature infants, enabling the use of standardized reference values for nerve examination.

More research is needed to confirm these findings for larger populations.

One of the strengths of this study is that it is the first to use high‐resolution ultrasound imaging to analyze the maturation of the peripheral nervous system in preterm‐born children. The results demonstrate that this examination method can serve as a straightforward diagnostic tool for premature infants as well. Additionally, this study is the first to establish conventional values of the median nerve for premature infants.

The limitations of this study include the fact that the age ranges of the study and control groups differ considerably, making it difficult to accurately compare the two groups. Additionally, ultrasound imaging has inherent error variability when estimating CSA, so we might not have been able to detect small differences between the groups.

Furthermore, there could be developmental differences between premature and full‐term infants related to other nerves, as indicated by the differing effect sizes for different cross‐sections by gestational age group.

Nevertheless, this study provides further insight into the development of the peripheral nervous system of premature infants. No significant differences were found between premature and full‐term infants with regard to the development of the peripheral nervous system. The association between CSA and BSA, as well as the logarithmic relationship between these values, was illustrated, which corroborates the findings of other studies (Jenny et al. 2020). BSA was found to be a better predictor of CSA than age. These findings suggest that, in the future, high‐resolution ultrasound imaging could be used to detect peripheral neuropathies and monitor the development of the peripheral nervous system in premature children, who are especially vulnerable to developmental problems.

## Author Contributions

**Lynn Jansen**: Investigation, Data Curation, Writing – Original Draft, Visualization. **Noé Bürke**: Investigation, Data Curation. **Erin West**: Formal analysis, Software, Writing – Review & Editing. **Janina Wurster**: Investigation. **Philip Broser**: Conceptualization, Writing – Review & Editing, Supervision, Funding acquisition, Methodology, Project administration.

## Peer Review

The peer review history for this article is available at https://publons.com/publon/10.1002/brb3.70954.

## Supporting information



Supplementary 1: Scatter plot of the increase in CSA with age from conception in premature (red) and full‐term (blue) infants showing an overlapping logarithmic increase in both groups.

Supplementary 2: Scatter plot of the increase in BSA with age from conception showing the dependancy between the two variables.

## Data Availability

Data is available upon request.
