# Supplementary figures and images for: Analysis of the Maturation of the Median Nerve in Preterm‐Born Children During the First 3 Years of Life Using High‐Resolution Nerve Ultrasound Imaging
Source: Brain Behav. 2025 Oct 1;15(10):e70954. doi: 10.1002/brb3.70954 (PMC12484708; doi:10.1002/brb3.70954)

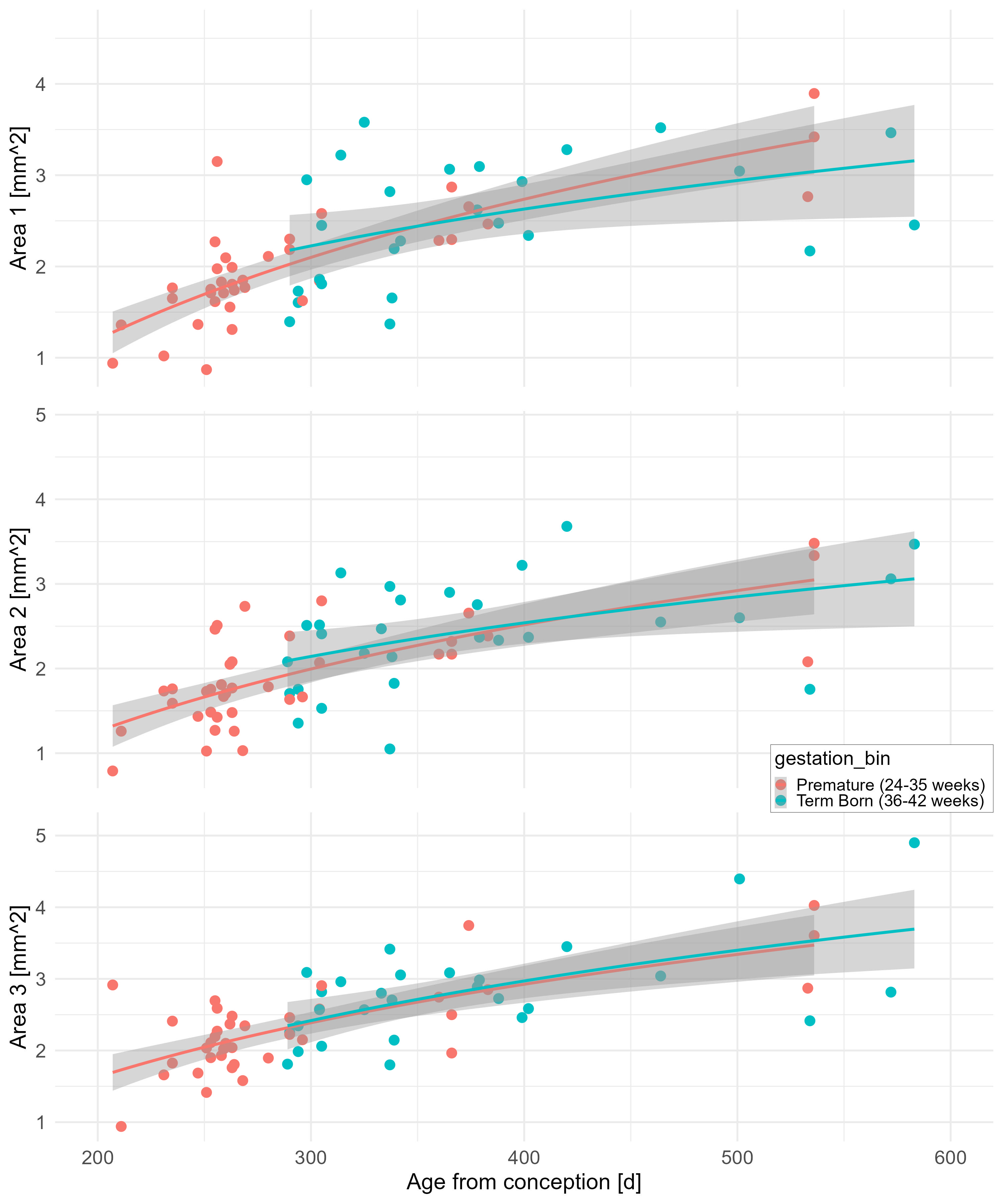

Supplement: Supplementary file 1 — Supplementary 1: Scatter plot of the increase in CSA with age from conception in premature (red) and full‐term (blue) infants showing an overlapping logarithmic increase in both groups. [file BRB3-15-e70954-s002.jpeg]

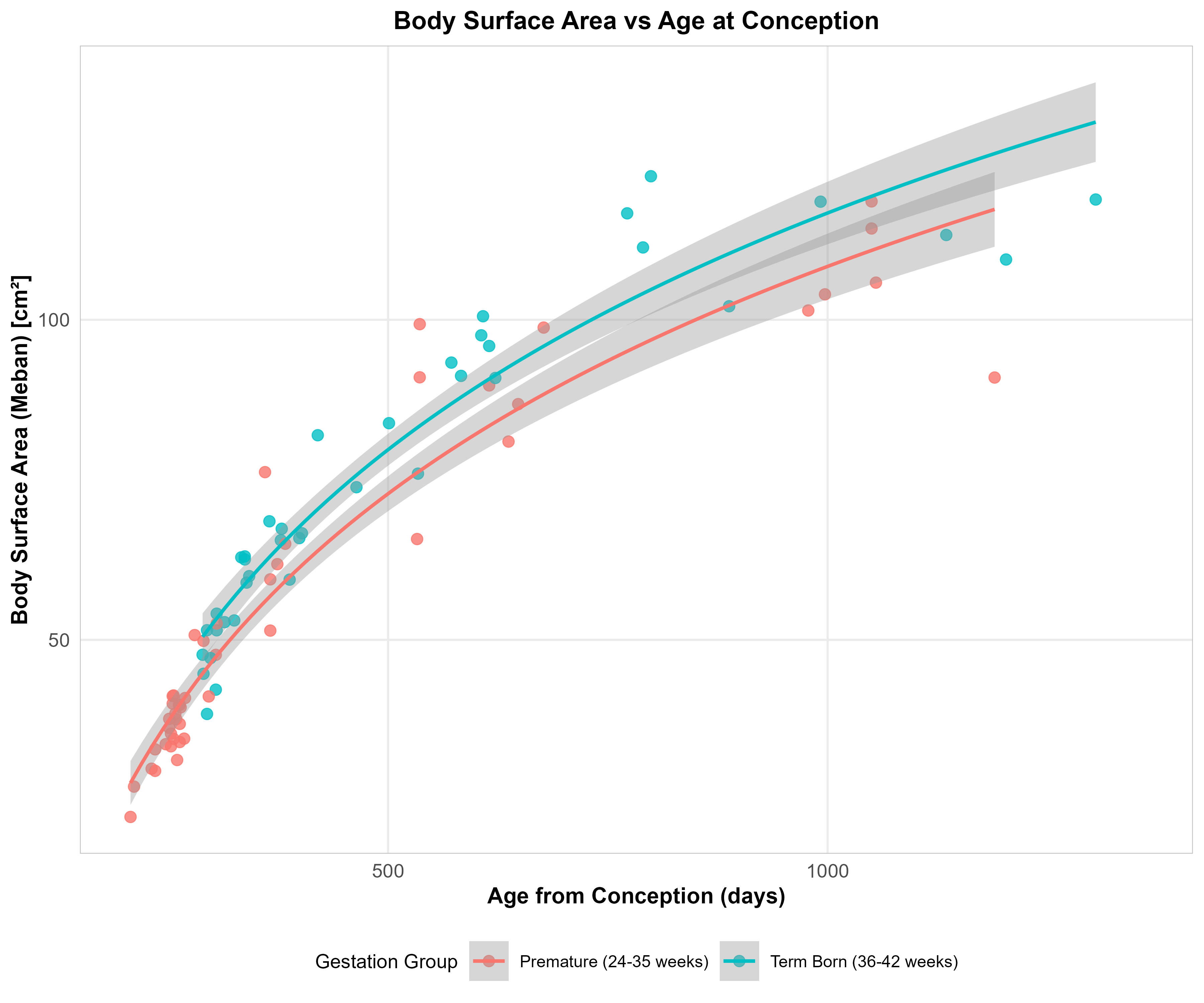

Supplement: Supplementary file 2 — Supplementary 2: Scatter plot of the increase in BSA with age from conception showing the dependancy between the two variables. [file BRB3-15-e70954-s001.jpeg]
